# Uniting disciplines against antimicrobial resistance (AMR): highlights from a multidisciplinary inaugural AMR summit

**DOI:** 10.1017/ash.2025.10039

**Published:** 2025-07-11

**Authors:** Brittany Rodriguez, Andrea M. Prinzi, Brandon K. Hill, Robert Tibbetts, Heinz Salazar, David McAdams, Awilda M. Rivera-Acosta, Minkey Wungwattana, Suzane Silbert, Denver T. Niles

**Affiliations:** 1 Department of Pharmacy Services, Texas Children’s Hospital, Houston, TX, USA; 2 US Medical Affairs, bioMérieux, Salt Lake City, UT, USA; 3 Microbiology, Henry Ford Health, Detroit, MI, USA; 4 Microbiology, Tampa General Hospital, Tampa, FL, USA; 5 Fuqua School of Business and Economics Department, Duke University, Durham, NC, USA; 6 Department of Pathology-Microbiology, Texas Children’s Hospital, Houston, TX, USA; 7 Baylor College of Medicine, Department of Pathology, Houston, TX

## Abstract

Antimicrobial resistance (AMR) poses a significant global health threat, projected to cause 10 million deaths annually by 2050. Addressing AMR requires a coordinated, multidisciplinary approach encompassing infectious disease (ID) clinicians, pharmacists, microbiologists, infection preventionists, and policymakers. The inaugural AMR Summit, hosted by bioMérieux in collaboration with Tampa General Hospital and the University of South Florida Morsani College of Medicine in November 2024, convened experts from various fields to explore innovative strategies for combating AMR. Key topics discussed included the role of multidisciplinary teams in antimicrobial stewardship programs, advancements in rapid diagnostic tests and antimicrobial susceptibility testing, the application of implementation science in AMR, and the integration of next-generation sequencing in ID diagnostics. The summit underscored the importance of diagnostic innovation, interdisciplinary collaboration, policy, advocacy, and public engagement in advancing efforts against AMR.

## Introduction

In December 2014, researchers from the United Kingdom published a pivotal study forecasting that antimicrobial resistance (AMR) could cause 10 million deaths annually by 2050, surpassing all other global health problems.^1^ This landmark analysis brought AMR to the forefront of public and scientific discourse, highlighting its potential to become the most significant public health crisis of our time. More recently, a 2024 study revealed that over 1 million deaths were attributed to AMR between 1990 and 2021.^2^ Using predictive modeling, the study estimated that over 39 million lives could be lost to AMR from 2024 to 2050. These findings underscore the urgent need for coordinated, multidisciplinary efforts to mitigate the growing impact of AMR.

**Figure 1.**
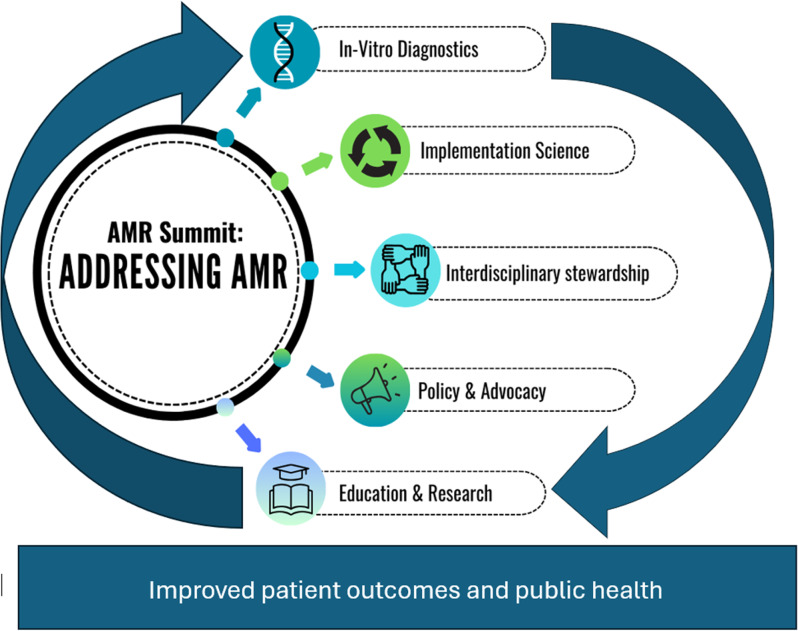
Key elements identified from the antimicrobial resistance (AMR) summit for addressing AMR.

Addressing AMR requires coordinated efforts across clinical and non-clinical sectors, leveraging innovative approaches to infectious diseases (ID) diagnostics, impact assessment, and scientific communication (Figure 1). Superheroes leading the fight are ID clinicians and pharmacists, clinical microbiologists, and infection preventionists (IP). The clinical microbiology laboratory is a core component of the antimicrobial stewardship program (ASP) efforts, helping clinicians answer three critical questions: Is the patient’s condition caused by an infectious pathogen? If so, what pathogen is responsible? Which antimicrobial(s) are optimal for treatment? Advances in diagnostic technologies have enhanced the ability to answer these questions, particularly for bloodstream infections (BSIs).^3–5^

Rapid diagnostic tests (RDTs), when paired with active ASP interventions, have substantially improved patient outcomes and healthcare costs.^6–8^ A recent systematic review and meta-analysis highlighted that RDT plus ASP workflows significantly reduced time to optimal therapy, length of stay (LOS), and mortality rates for BSIs compared to standard blood culture methods.^5^ While these data are promising, widespread adoption of RDTs and ASPs requires advocacy, strategic business planning, and leadership support. For example, a 2017 study by Goff et al. highlighted how involving hospital administration in ASP mentorship programs led to increased funding for RDTs and protected time for ID pharmacists and clinicians to focus on ASP activities, emphasizing the importance of demonstrating quality, clinical, and economic outcomes to support integration into practice.^9^

Sustained progress in AMR management also requires government and global collaboration. In the U.S., the National Action Plan for Combating AMR prioritizes RDT adoption, while the Centers for Disease Control and Prevention (CDC) provides guidelines for developing and maintaining ASPs. Internationally, organizations like the Foundation for Innovative New Diagnostics and the World Health Organization connect stakeholders, fund diagnostic innovations, enhance AMR surveillance, and advocate for policy changes.^10–12^ Building public support for ASPs and diagnostics requires innovative communication strategies such as patient advocates who can share compelling stories about their impact. Training for future ID and microbiology professionals should emphasize a one-health approach, fostering global awareness of AMR/ASP and skills to effectively communicate with diverse audiences, from the public to policymakers.^13,14^

In November 2024, bioMérieux hosted its inaugural AMR Summit in Tampa, Florida, to address the challenges and identify solutions related to AMR. In collaboration with Tampa General Hospital, a bioMérieux Center of Excellence, and the University of South Florida Morsani College of Medicine, the summit convened approximately 90 national experts, including policymakers, ID clinicians and pharmacists, microbiologists, patient advocates, industry and professional society leaders, and health economists. Attendees shared expertise and explored strategies to accelerate progress in addressing AMR and promote coordinated action. Key topics discussed at the summit included:

1. The role of multidisciplinary teams in AMR and ASP programs
2. Policy, payer, and clinical practice intersections
3. Reducing AMR through implementation science
4. Advancements in antimicrobial susceptibility testing
5. Applications of next-generation sequencing
6. Call to action to address research, education, and policy gaps

## The role of multidisciplinary teams in AMR

The growing threat of AMR necessitates a coordinated, multidisciplinary approach, with ASPs serving as a critical frontline defense. Antimicrobials stewardship (AMS) includes a variety of targeted strategies aimed at promoting the responsible use of antibiotics for both therapeutic and preventive purposes.^15^ The effective implementation of an ASP depends on a strong collaboration among ID clinicians, ID pharmacists, microbiologists, and IP specialists^16–19^ This interdisciplinary teamwork is essential for fostering robust leadership support, which is vital to the success of the ASP. At the AMR Summit, the ASP team from Tampa General Hospital presented on the contributions of various departments in the fight against AMR. Below is a summary of the discussion that involved the microbiology laboratory, ID pharmacy, and IP teams.

While all ASP team members contribute to combating AMR, the microbiology laboratory, despite its critical role, is often underrepresented. Rapid diagnostic results are essential for guiding appropriate antibiotic use and supporting stewardship initiatives. RDTs have significantly reduced turnaround times, enabling earlier intervention and targeted treatment.^15,20–24^ Beyond its diagnostic contributions, the laboratory serves as a surveillance hub, detecting emerging resistance patterns, clusters of infections, and novel multidrug-resistant organisms. The laboratory’s expertise in pathogen detection is indispensable for infection control and outbreak management.

ID pharmacists closely collaborate with the microbiology lab on various facets of antibiotic testing and reporting, playing a pivotal role in treatment protocols and infection prevention strategies. Their oversight in antimicrobial use and susceptibility testing is essential in combating AMR. This collaboration has led to the development of targeted susceptibility comments for specific pathogens, enhancing antibiotic prescribing practices and reducing costs. Collaboration among ID pharmacists and ID physicians is essential particularly when addressing multifaceted patient needs and optimizing therapeutic outcomes. Insights from ID physicians can enhance the pharmacist’s approach to medication management and care strategies in challenging scenarios. This interprofessional dialog is vital for the comprehensive management of patients facing intricate health issues.

The IP team is a key partner in ASP accountability, bringing national best practices and ensuring regulatory compliance. With increased focus on hospital-acquired infections and their implications for Centers for Medicare & Medicaid Services (CMS) funding, the alignment of IP strategies with organizational quality metrics is crucial. Therefore, establishing a strong collaborative relationship between the ASP and IP teams is essential.^25,26^ The COVID-19 pandemic highlighted the need for adaptive infection control measures and strengthened collaborations between stewardship and IP teams.^27^ Furthermore, there is an increasing emphasis on incorporating nursing staff into ASP efforts. This integration presents a valuable opportunity to utilize nurses’ frontline insights and enhance the overall effectiveness of the program.^28^

Establishing and sustaining a successful interdisciplinary ASP faces challenges including program launch, identifying key priorities, ensuring effective communication, and securing adequate staffing. Realistic objectives build early success and a data-driven business strategy demonstrates cost savings and quality improvements. Stewardship strategies must be tailored to each hospital’s unique environment. One example of this adaptability was highlighted at the summit in which a children’s hospital in Houston, Texas implemented rapid identification systems for gram-positive organism identification from blood cultures. Initially, the microbiology laboratory relayed results directly to bedside nurses; however, this approach often led to delays in physician notification or uncertainty regarding the appropriate course of action, resulting in inconsistent implementation of recommended interventions. To streamline the process, results were instead relayed to ID pharmacists who were equipped with pagers to receive and effectively communicate rapid identification results. This modification significantly reduced time to optimal antibiotic therapy.

Building trust and collaboration with key stakeholders requires sustained effort and a long-term commitment. Effectively showcasing the clinical impact of RDTs requires strategic data utilization and consistent communication of key findings and updates to testing algorithms. This endeavor is often hindered by delays in data collection and interpretation, staffing limitations, fluctuations in patient demographics, and institutional changes. Regular interdisciplinary meetings are essential for maintaining transparency, providing updates, and evaluating new test methods or processes.

Despite the progress in fostering multidisciplinary collaboration, it is important to continuously explore innovative strategies to optimize the communication processes. Ongoing commitment to advancing patient care and streamlining laboratory operations is reflected in these improvement initiatives. Addressing challenges and fostering trust among team members are vital to ensuring success and sustainability of ASP initiatives. Prioritizing open communication and collaboration not only enhances the team’s effectiveness but also lays the foundation for long-term impact of these antimicrobial stewardship efforts.

## Intersection of policy, payers, clinical practice

RDTs are integral to improving clinical outcomes and combatting AMR, but their effectiveness hinges largely on clinician adoption and payor reimbursement. Increasing RDT use requires a collaborative approach involving healthcare providers, payors, and policymakers. This process often involves generating robust evidence that elucidates the clinical utility^29^ and economic value of RDTs, developing and disseminating clinical guidelines,^30^ and active advocacy to influence payor policies while securing the necessary funding.^31–34^ Clinicians are ideally suited to conduct research validating the efficacy of RDTs and, where possible, collaborate with manufacturers on test development and integration.

Clinicians contribute significantly to guideline development committees, which evaluate evidence and recommend test strategies. Establishing high-quality evidence and comprehensive guidelines is critical for shaping payor policy decisions. Chief medical officers (CMOs) within payor organizations may be receptive to conversations with representatives from diagnostic companies and clinical advocates, presenting an avenue to influence payor perspectives based on current data.

Legislative action is equally important in the fight against AMR. Clinicians, scientific organizations, and patient advocates can collaborate to influence policymakers and support initiatives like ASPs, public health surveillance, antibiotic prescribing regulations, and research funding. A policy publication from the Infectious Diseases Society of America (IDSA) outlines several recommendations for combating AMR, which include implementing economic incentives for antibiotic research and development, streamlined antibiotic approvals, enhanced federal coordination, strengthened surveillance, improved infection control, expanded support for rapid diagnostics, workforce training, and eliminating inappropriate antibiotic use in agriculture.^33^

A panel discussion at the AMR summit involving clinicians, payers, and government officials explored critical issues related to test reimbursement, policy alignment, and clinical needs. Table 1 summarizes key insights from this discussion, highlighting opportunities for collaboration, barriers to adoption, and strategies to enhance market access for rapid diagnostic tests. Stronger stakeholder partnerships are critical to overcoming current obstacles and maximizing the impact of RDTs.

**Table 1.** Discussion panel perspectives

	Critical Care Clinician	Payor	Policymaker (elected)
**Clinical utility**	• RDTs improve outcomes for conditions like sepsis, which have high morbidity, mortality, and cost of care. • Clinical utility of RDTs varies based on type of RDT and number of targets it assesses.	• Clinically significant conditions such as sepsis and pneumonia justify coverage if utility is demonstrated. • Clinical utility varies based on RDT type targets.	• Collaborative forums, like the AMR Summit, are needed to facilitate important discussions, emphasize the importance of ID, and the clinical utility of RDTs.
**Rapid diagnostic test utilization**	• RDTs are underutilized compared to conventional testing. • Slow implementation and adoption of these RDTs is due, in part, to limited reimbursement, particularly in EDs. • RDT use is lacking in EDs and outpatient settings, particularly in pediatrics	• RDTs lacking clinical utility data are overutilitized. • Smaller “panels” (ie, fewer targets) have better insurance coverage, while the clinical utility of large panels is less clear and often not covered.	• The outcome of a bill may hinge on whether policymaker(s) choose to impose mandates on payors. • Patient advocacy stories can significantly influence support for new technologies and therapies.
**Guidelines**	• Clear guidelines are needed for the use of small versus large panel RDTs.	• Payors require evidence and evidence-based guidelines to support coverage. New data provided by medical champions may drive change.	• Guidelines are an important factor in rational decision-making regarding payment practices and related policy issues.
**Diagnostic stewardship**	• Clinical team members (eg, ID pharmacists) need more oversight of RDT use.	• Diagnostic stewardship is important for determining which populations will benefit the most.	
**Antibiotic stewardship**	• The absence of RDT results can lead to suboptimal care and unnecessary antibiotic prescribing.	• HEDIS measures are essential for evaluating quality and supporting coverage. • Payors can support educational initiatives for smaller clinician groups by providing toolkits and facilitating data sharing.	• Policymakers can offer funding and resources to support ASPs • Policymakers can collaborate with healthcare professionals, patients, and other stakeholders to tailor ASPs to meet the needs of local communities.
**Action items**	• A need for clinician-led development of standardized reporting for RDTs. • A need for RDTs to be integrated into sepsis bundles.	• Clinical, academic, and industry partnerships should generate clinical utility data to support coverage. • Member education on payor role is needed • Incremental data or a proof of concept is sufficient for payor consideration. • RDT manufacturer(s) and dedicated clinicians need to present data to payor chief medical officer(s) to drive change.	

ASP, antimicrobial stewardship program; ID, infectious diseases. HEDIS; Healthcare Effectiveness Data and Information Set; RDT, Rapid diagnostic test; EDs, Emergency Departments.

## Reducing AMR through implementation science

Leonardo da Vinci’s observation that “everything connects to everything else” highlights the complex nature of human behavior, a critical factor in addressing AMR. A major contributor to AMR is the inappropriate use of antimicrobials. Antibiotic prescribing is a social act,^35^ serving as a tool clinicians use to navigate patient demands, manage diagnostic uncertainty, and conclude clinical encounters.^36^ Social determinants, including clinician-patient dynamics and factors like risk, anxiety, fear, emotional responses, and misperceptions, influence prescribing behaviors. Understanding these complexities is crucial for tackling antibiotic overuse and AMR.

While valuable, ASP interventions such as direct education, preauthorization, and audit and feedback are insufficient. Research demonstrates that direct educational initiatives often fail to result in sustained improvement, and policies can be circumvented.^37–41^ Developing interventions and technologies without considering socio-behavioral context reduces their impact, delays implementation, and compromises sustainability. Integrating research into practice is a slow process, with only 14% of research translated into clinical practice or evidence-based interventions (EBIs) within 17 years.^42–44^ Recognizing these timelines and barriers is essential for achieving meaningful change in the fight against AMR.

Implementation science (IS) offers a solution by focusing on methods to enhance the adoption and fidelity of EBIs in practice, bridging the gap between evidence and application. While not new to medicine, IS is a relatively emerging field within ASP and diagnostic stewardship. It begins by identifying the EBI—such as novel diagnostic tool, an electronic medical record alert, or a training program designed to mitigate inappropriate prescribing or optimize patient outcomes.^45^ Implementation strategies involve deliberate actions to promote EBI adoption, execution, and sustainability. Several theories, models, and frameworks guide this process. Progress models describe the translational research journey, while determinant frameworks, classic theories, and implementation theories provide insights into factors influencing implementation outcomes. Evaluation frameworks assess the effectiveness of the implementation process.^46^ These validated tools standardize concepts and definitions, enhancing generalizability and applicability for others aiming to refine their practice.^47–49^

This use of models and frameworks distinguishes IS from quality improvement (QI) efforts. IS aims to generate generalizable insights applicable in various settings, while QI efforts typically focus on a single healthcare center and are highly contextualized.^50^ However, elements of both can be combined in ASP projects, facilitating the adaptation of a larger-scale EBI to the local context of a single institution or disseminating QI findings to broader settings.^50^ For example, Claeys et al., used a determinant framework with qualitative methods to understand local practices and perceptions of potential diagnostic stewardship interventions for urinary tract infections (UTIs) within US Veterans Affairs medical centers.^51^ Their findings revealed barriers and facilitators that can inform tailored ASP interventions for increased adoption and sustainability.

Effective implementation is essential not only for designing interventions but also for optimizing clinical outcomes in research, particularly in the realm of in-vitro diagnostics. Outcomes such as prescribing rates are influenced by the diagnostic test itself and by human behaviors involving patients, providers, and healthcare teams. A test’s efficacy depends on its adoption; otherwise, its impact on outcomes remains unclear. Optimizing behaviors related to test collection, reporting, interpretation, and prescribing is crucial for achieving significant results with advanced ID diagnostics. Similar to standalone ASP interventions neglecting socio-behavioral dynamics, diagnostic tests alone are insufficient in combating AMR.^39^

The most vulnerable are often the most at risk for infectious diseases and AMR. Utilizing IS to comprehend local contexts may help mitigate these inequities. Integrating bioethical inquiry into ASP and IS ensures that populations are appropriately served.^52–55^ The risks and benefits of any AMR-focused intervention should be evaluated, considering alignment with local needs and priorities, stakeholder identification, community impact, and the initiative’s effect on the community and healthcare system.^56,57^ Given that prescribing and diagnostic practices are shaped by local context, social influences, and inherent biases, IS provides a more equitable framework for addressing the complex challenges of AMR.

## Advancing antimicrobial susceptibility testing

The availability of rapid antimicrobial susceptibility testing (rAST) is crucial for optimizing management and improving patient outcomes, particularly in sepsis. While traditional phenotypic AST methods require 48 to 96 hours, rAST can deliver results within 6 to 24 hours, enabling more targeted treatment and improved outcomes.^58,59^ Studies on rAST have yielded mixed results.^60^ Although most studies demonstrate faster reporting times and some treatment adjustments, they often fail to show significant improvements in mortality or length of stay (LOS), likely due to small sample sizes and limited statistical power.^59^ Studies integrating rAST with an ASP have demonstrated significantly improved outcomes,^60^ a finding corroborated by a meta-analysis that showed a substantial reduction in LOS and mortality when rapid diagnostics were coupled with ASP.^61^ The AMR summit discussions highlighted key implementation challenges, focusing on laboratory workflow logistics (including cost, testing scope, and reporting) and the crucial role of collaborating with ID pharmacists and other stakeholders for improved AMS.

Implementing rAST in the laboratory presents several complexities.^62^ Decisions regarding making testing universally available versus targeted application of testing, types of specimens to test, replacement of existing methods, and 24/7 availability versus centralized or batched testing must be addressed. Most hospitals prioritize rAST directly from positive blood cultures due to the critical nature of bloodstream infections, where timely antimicrobial optimization significantly impacts patient outcomes.^63,64^ This focus ensures rapid identification of effective therapy, helping to reduce mortality and improve ASP efforts. Laboratories must choose between phenotypic and genotypic AST, each with advantages and limitations. For example, while some resistance genes (eg *mecA, vanA/B*) reliably predict phenotypic outcomes and inform treatment, others (eg A*mpC, OXA*-48) pose challenges due to inducibility and variable AST profiles.^59^

The financial burden of rAST implementation remains one of the most significant obstacles. Beyond the initial cost of new testing platforms, laboratories must allocate considerable resources and time to validate assays per the Clinical Laboratory Standards Institute guidelines.^65,66^ Given these constraints, it is crucial for microbiology laboratories to collaborate with ID and ASP teams to optimize implementation strategies and maximize clinical impact.

A well-functioning ASP is integral to the success of rAST. A significant barrier to ASP interventions is the lack of ASP pharmacists to act on results in real-time.^8^ The impact of rAST is limited without timely reporting, communication, and action.^59^ A quality improvement project conducted by Texas Children’s Hospital, one of the participating sites at the Summit, reinforced this idea, demonstrating that direct communication of rAST results to an ASP pharmacist reduced time to appropriate vancomycin de-escalation from 44 to 4 hours.

AMR is a critical global health problem that can be addressed through faster and more accurate AST, enabling timely adjustments to antimicrobial therapy. While the benefits of rAST coupled with ASP are well supported, implementation requires careful planning to overcome logistical, financial, and communication barriers. Successful implementation depends on interdisciplinary collaboration among ASP/ID pharmacists, physicians, nurses, IP specialists, and microbiologists. The 2024 US Antibiotic Awareness Week theme “Fighting Antimicrobial Resistance Takes All of Us,” aptly captured this shared responsibility.^67^

## Future directions in NGS

Next-generation sequencing (NGS) technology has transformed healthcare, significantly impacting cancer research and care,^68^ rare disease identification, and personalized medicine. While its applications in clinical microbiology and ID continue to expand, the full potential of NGS in these fields is yet to be realized. ID diagnostics still primarily rely on conventional microbiology methods, despite the advances in molecular technologies. NGS holds considerable promise in four key areas of ID medicine: organism identification, bacterial and viral genotyping,^69^ AMR detection, and epidemiological surveillance.^70,71^ Traditionally, organism identification has relied on time-consuming methods, such as biochemical tests. The introduction of mass spectrometry has notably improved turnaround times for these tests. Additionally, molecular diagnostics, including laboratory-developed tests, have revolutionized microbial identification. Nonetheless, challenges persist, particularly with slow-growing organisms, such as *Mycobacterium* and other fastidious bacteria, which often lead to delayed identification. NGS has the potential to dramatically reduce the turnaround time for identifying these challenging pathogens.^72,73^ When implemented effectively for direct-from-specimen use in early patient care, NGS could also lower overall healthcare costs.^74^

Genotyping bacterial and viral pathogens provides valuable insights into treatment strategies, vaccine development, and infection severity, particularly through the detection of virulence factors like toxin production.^75^ One emerging area of interest is using NGS to identify drug-resistant tuberculosis, a disease that poses significant treatment challenges due to its complex regimens, drug interactions, side effects, and the need for prolonged therapy.^76^ Accurately identifying resistance mutations can lead to personalized treatment plans, ultimately enhancing patient outcomes. Furthermore, NGS plays a crucial role in detecting and preventing outbreaks, especially for communicable diseases like tuberculosis.^77^ Recent research has shown that combining NGS methodologies with machine learning applied to electronic health records can enhance outbreak detection methods in hospital settings, supporting infection prevention efforts and significantly reducing costs associated with outbreaks.^78^ On a global level, NGS offers the opportunity to improve AMR surveillance comprehensively, but this requires collaboration between high-income and low-income countries.^79^ Additionally, accessible reference databases that include genetic data and metadata, formed through inter-regional and international collaborations, are essential to fully realize the potential of NGS for surveillance.^79^

While AMR detection with NGS in routine clinical microbiology practice is feasible, it requires comprehensive databases or the simultaneous use of multiple databases. Targeted or enrichment approaches via direct-from-specimen NGS, such as in blood cultures, will also be required for effective AMR detection.^74^ Despite its potential, several challenges have prevented NGS from becoming a routine tool in clinical microbiology. Cost remains a major limitation, making NGS inaccessible to many laboratories. The technology requires significant preparatory work, and interpreting results requires a high level of expertise to translate data into actionable clinical decisions.^80^ For NGS to be adopted for routine use, quality control, guidelines, user-friendly bioinformatics pipelines, validation, and data storage infrastructure are necessary.^81^ Even with user-friendly informatics software, clinical and microbiological expertise is required to determine the significance of detected organisms. Despite these obstacles, the future of NGS in ID diagnostics appears promising. Realizing its full potential will require collaboration between laboratory professionals and clinicians to overcome challenges and integrate NGS into routine clinical practice.

## Call to action

The escalating threat of AMR demands decisive, coordinated action. Failure to act with urgency and collectively will have severe repercussions for healthcare systems and communities globally. The AMR Summit confirmed that we have the knowledge and the tools to make significant progress against this global threat. However, sustained success hinges on interdisciplinary collaboration, advocacy for supportive policies, and adequate resources. We urge healthcare providers, policymakers, researchers, and the public to unite to advance RDTs, optimize ASPs, and promote responsible antibiotic use. Each of us has a role to play in this fight. We call upon every individual and organization invested in the fight against AMR to consider the initiatives outlined in Table 2. The time to act is now. By uniting across disciplines and committing to collaboration, we can mitigate the threat of AMR and protect the health of current and future generations.

**Table 2.** AMR initiatives

Actions	Initiatives	Description
**Engage Actively in ASPs**	• Actively participate in ASPs designed to optimize antimicrobial use. • Advocate for the adoption of RDTs.	• Healthcare facilities can monitor prescription practices, educate clinicians on responsible antibiotic use, and streamline treatment protocols. • RDTs enable rapid identification and targeted therapy, reducing antibiotic misuse.
**Foster Interdisciplinary Collaboration**	• Break down silos within the healthcare sector.	• ID clinicians, pharmacists, microbiologists, infection preventionists, and public health officials must work closely together to share insights and coordinate efforts. • Collaboration enhances the effectiveness of current strategies and promotes innovation through diverse perspectives.
**Promote Research and Education**	• Invest in research for new antimicrobial agents, diagnostic tools, and treatment protocols. • Train healthcare providers on the principles of stewardship.	• Healthcare institutions, universities, and government should prioritize AMR research and create educational programs aimed at increasing awareness and understanding of AMR among healthcare professionals and the public.
**Amplify Advocacy for Policy Change**	• Support local, national, and international organizations actively working to address AMR. • Engage with policymakers to prioritize AMR on health agendas.	• Support the National Action Plan for Combating AMR and advocate for legislative measures promoting infection control, research funding, and global collaboration. • Participation in these advocacy efforts can impact policy development and resource allocation.
**Participate in Professional Dialogs and Networks**	• Attend AMR-focused conferences, workshops, and summits to stay informed about the latest research, best practices, and strategies being implemented across the globe.	• Share insights, experiences, and challenges with peers, and seek collaboration on innovative approaches to combat AMR. • Networking leads to valuable partnerships and enhances our collective capacity to address AMR.

ASPs, antimicrobial stewardship programs; AMR, antimicrobial resistance; RDTs, rapid diagnostic tests; ID, infectious diseases.
