# Uniting disciplines against antimicrobial resistance (AMR): highlights from a multidisciplinary inaugural AMR summit – ERRATUM

**DOI:** 10.1017/ash.2025.10099

**Published:** 2025-09-16

In the initial publication of this article,^1^ authors Suzane Silbert and Denver T. Niles appeared in incorrect order in the author list. The list has been corrected.

The publisher apologizes for the error.
